# Editorial: Innovative Approaches in the Management of Bone and Joint Infection

**DOI:** 10.3389/fmed.2021.789092

**Published:** 2021-11-18

**Authors:** Tristan Ferry, Sébastien Lustig, Frédéric Laurent, Alex Soriano

**Affiliations:** ^1^Infectious and Tropical Diseases Unit, Croix-Rousse Hospital, Hospices Civils de Lyon, Lyon, France; ^2^Université Claude Bernard Lyon 1, Lyon, France; ^3^Centre de Référence des Infections Ostéo-Articulaires complexes (CRIOAc Lyon), Hospices Civils de Lyon, Lyon, France; ^4^Centre International de Recherche en Infectiologie, CIRI, Inserm U1111, CNRS UMR5308, ENS de Lyon, UCBL1, Lyon, France; ^5^Orthopaedic and Sport Surgery Unit, Hôpital de la Croix Rousse, Hospices Civils de Lyon, Lyon, France; ^6^Institut des Agents Infectieux, Hôpital de la Croix Rousse, Lyon, France; ^7^Department of Infectious Diseases, Hospital Clínic, IDIBAPS, Barcelona, Spain; ^8^University of Barcelona, Barcelona, Spain

**Keywords:** bone and joint infection, prosthetic-joint infection, phage therapy, bacteriophage, lysin, OSCAT, personalized medicine, suppressive antimicrobial therapy

Bone and joint infections (BJI) are one of the most difficult-to-treat bacterial infectious diseases. Its management is complex, and requires a multidisciplinary approach, from the diagnosis to the medico-surgical strategy. This Research Topic brings together several breakthrough papers in microbiological diagnosis, personalized common or new therapies, and prevention of superinfection (Figure 1).

**Figure 1:**
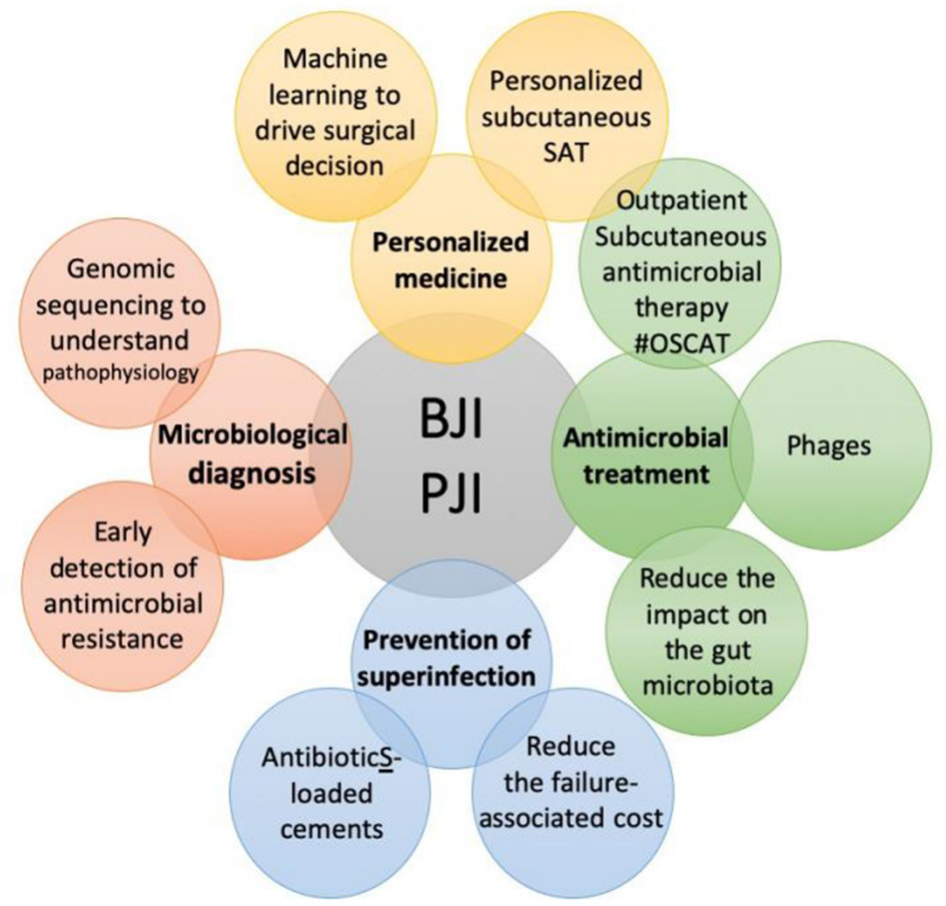
The specific themes covered in the Research Topic “Innovative Approaches In The Management Of Bone and Joint Infection”.

At present, diagnosis is easy for the acute forms, while most of the chronic infections remain undiagnosed or are discovered too late, leading to catastrophic clinical situations. Pham et al. reported a case where genomic analysis was decisive for the best therapeutic strategy, in a patient who experienced two episodes of *Streptococcus dysgalactiae subsp. Equisimilis* prosthetic-joint infection (PJI), that were finally unrelated. To improve the diagnosis of low-grade PJIs, Kolenda et al. showed that the treatment of an explanted prosthesis with dithiothreitol (a chemical agent that disrupts biofilm) used in a particular device could improve the microbiological diagnosis, by shortening the duration of cultures, or by identifying additional pathogens. This could provide an alternative to sonication, which is not so easy to implement and requires significant technical time.

To treat BJI, different medico-surgical strategies are proposed, depending on the type of BJI. However, the rate of success remains very disappointing, especially for patients with implant-associated BJI. In such patients, finding a way to cure the infection without adverse body reactions such as organ failure, or loss of function, remains a challenge, and deciding the treatment strategy for a particular patient with a specific form of BJI, is mainly based on experience, rather than precision medicine and the patient's clinical health data. Wouthuyzen-Bakker et al. reviewed the importance of risk scores for debridement, antibiotics, and retention of the implant (DAIR) in patients with PJI, and discussed the potential of implementing machine learning (artificial intelligence) in identifying patients who are at the highest risk for failure of DAIR will be addressed. The ultimate goal is to maximally tailor and individualize treatment strategies and to avoid treatment generalization, as also proposed by Baldan and Sendi. In this way, outpatient parenteral antimicrobial therapy (OPAT) is a potential option in particular patients with BJI, as described in the paper by Ferry et al. Moreover, Goutelle et al. demonstrated in a case series that pharmacokinetic/pharmacodynamic dosage individualization of suppressive beta-lactam therapy administered by subcutaneous route only three times in a week (without need for a central catheter) was feasible, safe, and beneficial for particular complex patients with PJI.

Chauvelot et al. present new important data about the pathophysiology and treatment of Corynebacterium BJI, an often-neglected etiological agent of post-traumatic and/or post-operative BJI. This infection requires complex and collaborative medical-surgical management as it is associated with a poor prognosis, which is mostly driven by the initial surgical debridement. Furthermore, if biofilm formation did not appear as a pivotal physiopathological mechanism of Corynebacterium in BJI, bone cell invasion via the cellular β1 integrin allows the formation of an intracellular reservoir that leads to chronic infection.

The most important contributions to this Research Topic relate to non-common anti-infective agents, such as bacteriophages or lysins, that are new ways to specifically target the pathogen. They have antibiofilm activities and Ferry et al. report emergent approaches to keep the function in patients with chronic PJI for whom phages were administered during open debridement, during arthroscopy, or within a hydrogel in a patient with knee megaprosthesis infection. Ferry et al. also reported the use of exebacase (CF-301), a lysin that targets staphylococci species, as salvage therapy in elderly patients with relapsing multidrug-resistant *S. epidermidis* prosthetic knee infection. These approaches probably improve the efficacy of suppressive antibiotics and prevent major loss of function, and clinical trials are now needed. These breakthrough papers have been viewed and are already largely cited, and will help to promote the creation of nation-wide phage therapy centers dedicated to implant-associated infections and other difficult-to-treat infections, especially if a multidrug-resistant bacteria are involved (1).

Finally, a better knowledge of the costs, risks, and consequences of the management of patients with chronic PJI is crucial. In the paper by Bourbotte-Salmon et al., patients with chronic total knee arthroplasty infection, requiring revision using rotating hinge implant, had good functional outcomes but experienced a high rate of septic failure, mostly due to bacterial superinfection. These patients need optimal antimicrobial systemic prophylaxis and innovative approaches to reduce the rate of superinfection. The cost of superinfection was evaluated in the paper by Serrier et al. This study revealed that chronic PJI requiring a 2-stage revision is costly, with significant costs arising from the reimplantation procedure (about 15 k€). However, following reimplantation, the rate of subsequent new infection remained high, and the cost of reimplantation following a new infection is considerable, reaching 50 k€ per patient. These first cost estimates of managing chronic PJI with 2-stage exchange in France underline the economic interest of preventing new infections. A one-stage approach for patients with chronic PJI, including patients with fistula, as described by Marmor et al., could be an option for reducing the cost attributed to superinfection attributable to the 2-stage approach, as the infection control rate at 2 years was 95.3% in patients treated in this monocentric study. However, generalization of one-stage procedure in this context could be associated with bacterial persistence, which needs to be prevented (2). In patients for whom cemented prosthesis is needed for septic revision is required, the use of commercially available bone cement, used for prosthesis fixation and loaded with gentamicin plus vancomycin or clindamycin, can help in preventing superinfection. Indeed the paper published by Cara et al. compared the prophylactic anti-biofilm activity of various commercial cements on different staphylococcal strains that are resistant to antibiotics, and found that cement eluting a combination of antibiotics had a significantly better ability to inhibit biofilm formation than the cement eluting only gentamicin. Finally, Benech et al. evaluated the potential adverse effect on the gut microbiota of systemic antibiotics used to treat patients with BJI. Systemic antibiotics significantly altered the gut microbiota diversity and composition, with a rapid but partial recovery observed at 2 weeks after antibiotic withdrawal. Antibiotic duration or the use of fluoroquinolones did not seem to affect this resilience. In this paper, the acquisition of multidrug-resistant bacteria carriage in the gut remained one of the most challenging side effects of long-term exposure to antibiotics. Innovative microbe-based therapies could be a promising tool to address these issues.

Taken together, each of the articles published in this Research Topic has contributes to improving the diagnosis, management, and outcomes of BJI. Promoting translational research in expert centers such as CRIOAc (3), the compilation of such studies in this Research Topic, aims to share experiences in multidisciplinary societies and congresses such as the European Bone and Joint Infection Society. These types of initiatives are crucial to innovate and considerably reduce the burden of BJI, which remains a neglected infectious disease in many industrialized countries.

## Author Contributions

TF initiated the Research Topic and wrote the draft of the manuscript. All authors approved the submission.

## Conflict of Interest

The authors declare that the research was conducted in the absence of any commercial or financial relationships that could be construed as a potential conflict of interest.

## Publisher's Note

All claims expressed in this article are solely those of the authors and do not necessarily represent those of their affiliated organizations, or those of the publisher, the editors and the reviewers. Any product that may be evaluated in this article, or claim that may be made by its manufacturer, is not guaranteed or endorsed by the publisher.

## References

[1] Pirnay JP Ferry T Resch G. Recent progress towards the implementation of phage therapy in Western medicine. FEMS Microbiol Rev. (2021) 21:fuab040. 10.1093/femsre/fuab040 34289033

[2] Bernard L Arvieux C Brunschweiler B Touchais S Ansart S Bru JP. Antibiotic therapy for 6 or 12 weeks for prosthetic joint infection. N Engl J Med. (2021) 384:1991–2001. 10.1056/NEJMoa2020198 34042388

[3] Ferry T Seng P Mainard D Jenny JY Laurent F Senneville E. The CRIOAc healthcare network in France: a nationwide Health Ministry program to improve the management of bone and joint infection. Orthop Traumatol Surg Res. (2019) 105:185–90. 10.1016/j.otsr.2018.09.016 30413338

